# Molecular hallmarks of neurodegeneration in polyglutamine spinocerebellar ataxias

**DOI:** 10.1038/s41419-025-08154-2

**Published:** 2025-11-10

**Authors:** Clévio Nóbrega, Adriana Marcelo, Ana Teresa Rajado, André Conceição, Bernardo Estevam, Carlos A. Matos, David VC Brito, Inês T. Afonso, José Miguel Codêsso, Rebekah Koppenol, Rafael G. Costa, Ricardo Afonso-Reis, Rodrigo Paulino, Tiago Moreira-Gomes

**Affiliations:** 1 Algarve Biomedical Center Research Institute (ABC-Ri), University of Algarve, Faro, Portugal; 2 Faculty of Medicine and Biomedical Sciences, University of Algarve, Faro, Portugal; 3 PhD Program in Biomedical Sciences, Faculty of Medicine and Biomedical Sciences, University of Algarve, Faro, Portugal

**Keywords:** Spinocerebellar ataxia, Cellular neuroscience

## Abstract

Polyglutamine spinocerebellar ataxias (PolyQ SCAs) comprise a group of six inherited rare neurodegenerative diseases. They are caused by abnormal mutation of a CAG tract in six otherwise unrelated genes, leading to a complex cascade of molecular events that culminate in neuronal death. Based on decades of research in these diseases, this review identifies and categorizes the distinctive hallmarks involved in the molecular pathogenesis of PolyQ SCAs. We organized these molecular signatures into three groups: (i) primary hallmarks, which directly refer to the transcription and translation of the abnormally expanded gene and protein, respectively; (ii) secondary hallmarks, which include alterations in pathways and organelles that are implicated in the disease pathogenesis; and iii) end-stage hallmarks, which highlight the final events of the pathogenesis cascade in PolyQ SCAs. This framework is expected to provide a platform for understanding the complex network of molecular mechanisms involved in these diseases and to guide current and future efforts in developing therapies.

## Facts

- In PolyQ SCAs, the abnormally expanded polyQ tract increases the propensity for the protein to self-assemble and aggregate into large intracellular multiprotein inclusions that are found in the nervous tissue of patients.
- The abnormal interaction of polyQ proteins with other molecules represents a toxic gain-of-function that impacts neuronal homoeostasis and contributes to toxicity and neurodegeneration.
- In PolyQ SCAs, polyQ proteins are targeted by posttranslational modifications (PTMs) that modulate toxicity of the disease-related proteins.
- Emerging evidence suggests that, in PolyQ SCAs, the expanded CAG repeat-bearing RNA itself plays a direct and detrimental role in the molecular pathogenesis of these disorders.
- Dysfunction of several pathways and cell organelles is implicated in the pathogenesis of PolyQ SCAs, including mitochondrial dysfunction, dysregulation of cellular degradation systems, transcriptional dysregulation, defects in signal transmission, and calcium signalling alterations.
- The end-stage hallmarks, neuroinflammation and neuronal death, arise as a conclusion of the pathogenic process.

## Open Questions

- What is the actual toxic role of protein aggregation observed in the pathological process?
- Could a loss-of-function of the interacting partners of polyQ proteins also contribute to the molecular pathogenesis of PolyQ SCAs?
- Which specific cellular processes are disrupted by the presence of the expanded RNA repeats, and how do they lead to cellular dysfunction and neurodegeneration?
- Is the dysregulation of particular cell pathways or organelles a cause or a consequence of other pathogenic events? How are the diverse elements contributing to PolyQ SCAs molecular pathogenesis interconnected?
- How to explain the regionally selective neuronal loss that is observed in PolyQ SCAs?

## Introduction

Spinocerebellar ataxias (SCAs) are a genetically heterogeneous group of autosomal dominantly inherited progressive disorders clinically characterized by a loss of balance and motor coordination accompanied by slurred speech. From a genetic point of view, SCAs fall into two major groups: those caused by repeat expansion mutations and those caused by non-repeat mutations [1]. In the former group, polyglutamine SCAs (PolyQ SCAs) represent a group of six diseases, encompassing SCA1, SCA2, SCA3 (also known as Machado-Joseph disease, MJD), SCA6, SCA7, and SCA17 [2–4]. These conditions result from the expansion of the CAG trinucleotide repeat within the coding regions of the causative genes, leading to the production of an abnormally long stretch of glutamines in the respective protein. In some patients, the repeat sequence is not purely CAG but may be interrupted by other triplets, such as CAA or CAT [5].

This abnormal expansion causes local conformational alterations in the protein, increasing its propensity to aggregate and leading to the formation of intracellular inclusions, which are one of the main features of these diseases [6]. It is currently accepted that dentatorubral–pallidoluysian atrophy (DRPLA) is classified as a PolyQ SCA [1]. However, considering that DRPLA exhibits features not found in other SCAs and the limited research on its molecular pathogenesis, we have not included it in this review [7].

SCA1, SCA2, SCA3/MJD, and SCA7 are caused by an abnormal expansion in the *ATXN1*, *ATXN2*, *ATXN3*, and *ATXN7* genes, respectively. Similarly, SCA6 is linked to abnormal CAG expansion in the gene encoding the subunit-α of the Cav2.1 voltage-gated calcium channel (CACNA1A) and SCA17 to mutantion of the gene encoding TATA-box-binding protein (TBP) [8]. Despite their heterogeneity, PolyQ SCAs share some clinical features, including a positive correlation between the number of CAG repetitions and the age at onset and severity of manifestations. Moreover, they all display genetic anticipation, wherein the size of the repeat expansion tends to increase across successive generations [3, 9].

Depending on the PolyQ SCA, the minimum number of CAG repetitions causing each disease varies [10]. Nevertheless, the existence of this minimum number of repeats highlights its central causative role (Table 1). While normally the abnormal expansion in polyQ proteins is a pure CAG tract, in SCA1 and SCA2, it was shown that CAA or CAT interruptions within the CAG repeat stretch reduce allele penetrance and increase meiotic stability [11, 12]. Although the expansion in the gene, mRNA, and protein are at the basis of the pathological alterations and clinical manifestations, several other molecular and cellular events contribute decisively to the establishment and progression of these diseases. The apparent simplicity of this “monogenic” group of neurodegenerative disorders contrasts with the fact that it is widely accepted that the molecular events underlying pathogenesis of PolyQ SCAs are multifaceted [13].

**Table 1:** Summarized features of polyglutamine spinocerebellar ataxias.

Disease	Gene	Protein	Normal repetitions	Disease repetitions*	Main affected brain regions
SCA1	*ATXN1*	Ataxin-1	6–38	39–44	Cerebellum; brainsteam; spinal cord
SCA2	*ATXN2*	Ataxin-2	14–31	35–500	Cerebellum; brainstem; *substantia nigra*
SCA3/MJD	*ATXN3/MJD1*	Ataxin-3	11–44	61–87	Cerebellum; basal ganglia; brainstem; *substantia nigra*
SCA6	*CACNA1A*	Cav2.1	4–18	20–33	Cerebellum; dentate nucleus
SCA7	*ATXN7*	Ataxin-7	4–19	36-460	Cerebellum; brainstem; basal ganglia; visual cortex
SCA17	*TBP*	TATA-binding protein	25–42	49–66	Cerebellum; inferior olive

*SCA1* Spinocerebellar ataxia type 1, *SCA2* Spinocerebellar ataxia type 2, *SCA3* Spinocerebellar ataxia type 3, *SCA6* Spinocerebellar ataxia type 6, *SCA7* Spinocerebellar ataxia type 7, *SCA17* Spinocerebellar ataxia type 17. * Full penetrance.

**Fig. 1:**
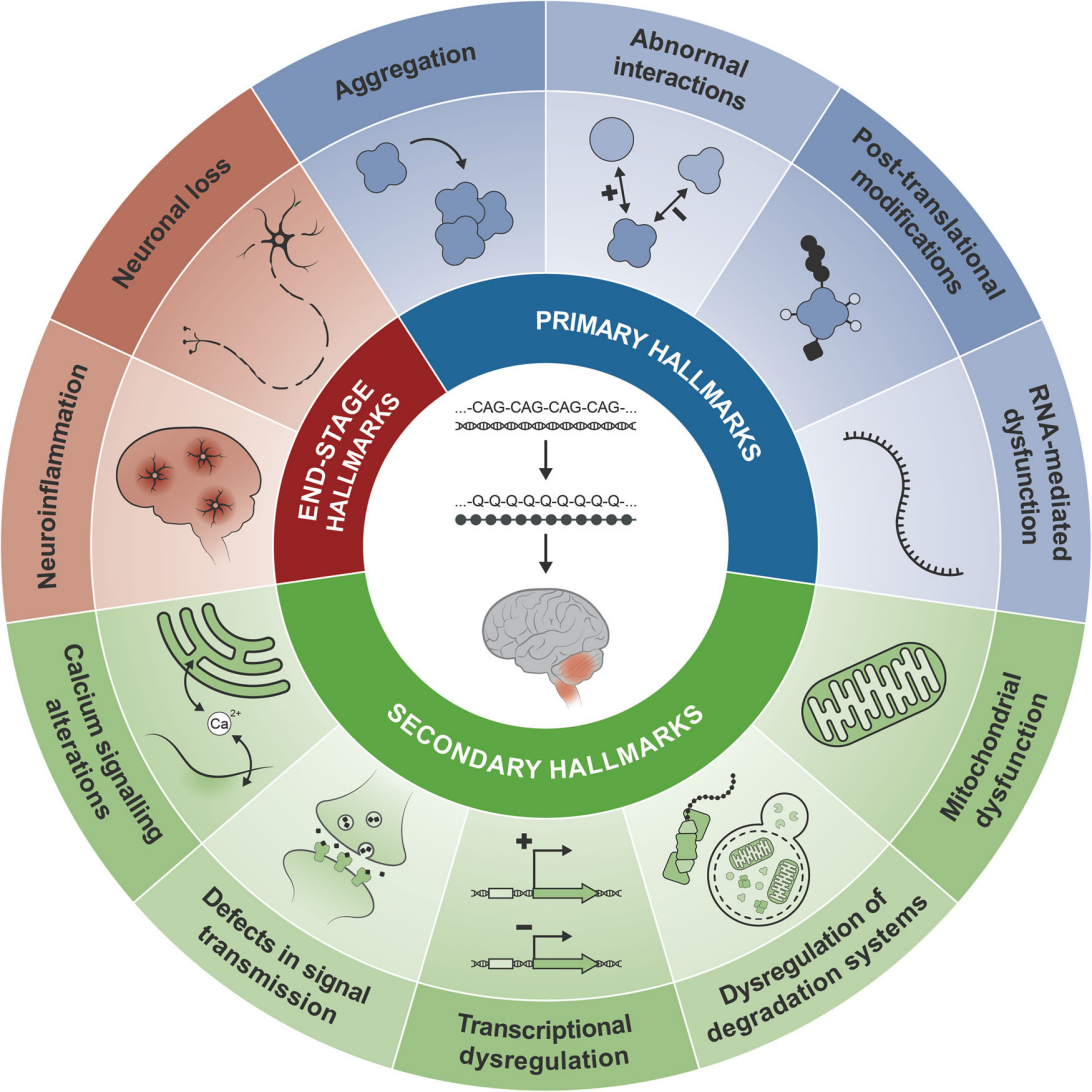
Molecular hallmarks of polyglutamine spinocerebellar Ataxias. Primary hallmarks, which directly refer to the transcription and translation of the abnormally expanded gene and protein, respectively; secondary hallmarks, which categorize pathways and organelles that are implicated in the disease pathogenesis; and end-stage hallmarks, which highlight the final events of the pathogenesis cascade in these diseases.

**Fig. 2:**
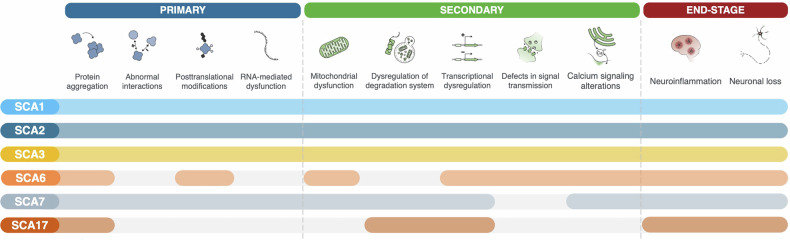
Molecular hallmarks involvement in each of the polyglutamine spinocerebellar Ataxias. For the most common PolyQ SCAs there are reports of the involvement of most of the hallmarks identified in this review. For others, like SCA6 and SCA17 there are still some hallmarks for which there is no study reporting its involvement; however, based in the common molecular pathogenesis mechanisms among PolyQ SCAs it is likely that all hallmarks are important in the disease.

In this review, we identify and categorize the distinctive hallmarks that are decisively involved in the molecular pathogenesis of PolyQ SCAs, similarly to the framework proposed for ageing, cancer, and other neurodegenerative diseases [14–16]. The hallmarks were identified based on their presence in at least two PolyQ SCAs and categorized into three groups (Figs. 1 and 2): (i) primary hallmarks, which directly relate to the transcription and translation of the abnormally expanded gene and protein; (ii) secondary hallmarks, which encompass cell pathways and organelles that have been described to be dysfunctional and contribute to disease; and (iii) end-stage hallmarks, which highlight the final events of the pathogenic cascade.

## Primary Hallmarks

### Aggregation

Proteins are essential for the proper functioning of cells, and their three-dimensional structures are crucial for their biological activity. Proteins are primarily folded during translation, and their amino acid sequence is a major determinant of their final conformation. However, various factors, such as genetic mutations, environmental stressors, or ageing, can lead to the improper folding of proteins [17]. Misfolded proteins associated with neurodegenerative diseases tend to self-assemble, forming insoluble aggregates and, in some cases, toxic soluble oligomers [18]. Aggregate-prone proteins may accumulate into large macromolecular inclusions that are associated with later stages of the pathological process [19]. Both smaller aggregates resulting from protein self-assembly and larger inclusions are thought to interfere with cellular processes and constitute one of the hallmarks of proteinopathies, which include neurodegenerative disorders like Alzheimer’s or Parkinson’s diseases.

In PolyQ SCAs, the abnormal PolyQ tract increases the propensity for the protein products of the CAG-expanded genes to aggregate and eventually form large intracellular multiprotein inclusions that are detected in the *postmortem* nervous tissue of patients [3, 20–25], constituting a major neuropathological mark [5].

1) The expanded PolyQ tract, which can be considered a major driver of the observed toxicity, leads to an increased propensity of polyQ proteins to aggregate forming neuronal inclusions. In SCA1, expanded ATXN1 was found to derive cellular toxicity, causing damage to neurons [26]. In SCA2, the impact of the toxic PolyQ repeat is less clear [27]. On one hand, increased PolyQ expansions in ATXN2 lead to heightened cellular toxicity. On the contrary, ATXN2 N-terminal fragments containing the PolyQ repeat are less toxic than the full-length protein [28]. Another study demonstrated that the *ATXN2* gene, when contains a pure CAG sequence in its PolyQ repeat domain, is linked to higher toxicity compared to a version interrupted by CAA/G sequences. The interrupted sequence did not exhibit the same toxic effects in *Drosophila* [29]. In SCA3/MJD, it is widely accepted that the expanded polyQ tract in the C-terminal region of ATXN3 leads to toxicity [30]. In SCA6, initial studies pointed out that PolyQ expansions in the causative protein led to a reduction in Ca^2+^ influx into Purkinje cells and other neurons, which led to the observed cellular death [31]. Later, it was shown that SCA6 causative C-terminal protein with 33 glutamines led to twice the rate of cell death as those containing unexpanded tracts [32]. In SCA7, it was shown that the expression of PolyQ-expanded ATXN7 results in an increase in reactive oxygen species (ROS) levels and later cellular toxicity [33]. These observations raise an important question: What is the actual impact of protein aggregates on the toxicity observed in the pathological process? Currently, the mainstream view is that the formation of large inclusions is not the cause of the toxicity observed in PolyQ SCAs, which could instead be driven by oligomers, proteolytic cleavage fragments, or even the expanded mRNA [2, 24, 34, 35]. Nevertheless, aggregation is still envisioned as an important deleterious process in these diseases [24, 36, 37]. Findings in *postmortem* patient brain tissue support the idea that inclusions may have a benign role, as their localisation in the brain does not appear to properly coincide with the regions that undergo degeneration [38]. In this view, inclusions are perceived as a reservoir for toxic species, sequestering them, preventing their harmful effects on neurons, and therefore, they might have a neuroprotective function.

2) Depending on the disease, the subcellular localisation of the pathological inclusions is different: in SCA1, SCA3/MJD, SCA7, and SCA17, they are mainly nuclear [30, 39–42], while in SCA6 and SCA2, they are cytoplasmic [43, 44], although there are some reports of nuclear inclusions in SCA2 patients [38, 45, 46]. What roles do the subcellular localisation of the abnormally expanded protein, and the formation of inclusions play in the pathogenesis? The impact of inclusions on neuronal function and the toxicity of the pathological process can differ based on their location within the cell. In fact, in SCA1, SCA3/MJD, and SCA7, the nuclear accumulation of the causative protein and inclusions was shown to be critical to toxicity [47–49]. In SCA2, the abnormally expanded ATXN2 alters its localisation, disrupts the normal morphology of the Golgi complex, and contributes to cell death [50]. In SCA3/MJD, inclusions were also observed in axons, which could impact axonal transport and neuronal homoeostasis [51]. The subcellular localisation of inclusions may reflect the toxic gain-of-function of the expanded protein. Moreover, given its potential role in disease progression, it is possible that changes in its subcellular localisation could be considered a hallmark of the final stages of PolyQ SCAs pathogenesis.

3) The pathological inclusions contain the causative PolyQ-expanded proteins and other sequestered proteins, which hamper their normal function, possibly contributing to pathogenesis [52–54]. In SCA1 patient samples and mouse models, the expanded ATXN1 protein abnormally interacts with DNAJ Heat Shock Protein Family (Hsp40) Member A1 (HDJ-2/ HSDJ), a chaperone, recruiting it into inclusions [55]. In SCA2 models, it was shown that the expanded ATXN2 recruits its interactor Poly(A)-binding protein cytoplasmic 1 (PABPC1), into inclusions, leading to a progressive insolubility of both proteins and enhancing the disease pathology [56]. In the same line, two interactors of ATXN2, the ubiquitin ligases F-Box and WD repeat domain containing 8 (FBXW8) and parkin RBR E3 ubiquitin protein ligase (PARK2), were shown to be driven into insolubility by expanded ATXN2 [57]. In SCA2 patients brain samples, several RBPs, such as TAR DNA-binding protein (TDP-43), and Staufen1 (STAU1), are sequestered to expanded ATXN2 aggregates [58–60]. In SCA3/MJD, similar observations were registered, as expanded ATXN3 abnormally sequesters proteins, such as valosin-containing protein (VCP), ubiquitin, and chaperones into inclusions [61–63]. Moreover, in SCA3/MJD, it has been observed that ATXN2 is recruited into inclusions in patient samples, and its protein levels are reduced in both patient samples and animal models [43, 64]. In SCA7, expanded ATXN7 sequesters a subunit of the deubiquitinase (DUB) module (DUBm) in the Spt-Ada-Gcn5 acetyltransferase (SAGA) complex, disrupting its normal function [65]. In the SCA7 cellular model and patient brain samples, several heat-shock proteins and proteasome subunits were found to be recruited into inclusions. Additionally, activated caspase-3 was also recruited into these inclusions and was upregulated in cortical neurons, suggesting that it may contribute to the disease process [41, 64]. Also, in SCA7 models, the RBPs TDP-43, Muscleblind-like splicing regulator 1 (MBNL1), and fused in sarcoma (FUS) are recruited to expanded ATXN7 aggregates [66].

4) The causative proteins of PolyQ SCAs, including their abnormally expanded forms, are expressed throughout the brain [46, 47, 67–72]. However, the formation of inclusions, which have an amyloid or fibrillar nature [73], mostly occurs in specific brain regions [74]. Moreover, their presence does not correlate with the regions where neurodegeneration is primarily observed. But why does the selective aggregation occur in specific neuronal populations? This selective aggregation could result from differences in protein expression levels, posttranslational modifications (PTMs), protein interactions, differences in intracellular environment, and even the ageing process, as it is linked to a dysregulation of protein quality control systems. It has been demonstrated that expressing the expanded *ATXN2* gene in aged animals worsens the SCA2-like phenotype when compared to expression in younger animals, leading to increased aggregate formation and a greater loss of neuronal markers [75]. Also, some neuronal subtypes of the cerebellum and brainstem may exhibit increased vulnerability to protein aggregation and neurodegeneration due to their specific features and functions [76]. In SCA2, cerebellar Purkinje cells are one of the most affected cell types, possibly because of the high expression levels of ATXN2 in these cells. In the non-repeat SCA28, the selective vulnerability of the cerebellum is consistent with the high levels of the protein causing the disease [77].

The aggregation of expanded PolyQ proteins and the resulting formation of inclusions are key features of neurodegeneration in PolyQ SCAs. However, more studies are needed to clarify the effective role of aggregation in the disease pathogenesis and the extent of the toxicity of the species that are formed in the process.

### Abnormal interactions

Protein-protein interactions are essential for nearly all molecular processes within the cell. They are particularly important in the brain because of the brain’s compartmentalized structure and its reliance on a highly interconnected molecular network [70].

1) In PolyQ SCAs, the expanded polyQ proteins lead to the establishment of aberrant interactions with other molecules. This represents a toxic gain-of-function that impacts the neuronal homoeostasis and contributes to neurodegeneration. A study showed that the toxic gain-of-function of the ATXN1-CIC (cognate partner capicua) complex is the major driver of toxicity in the cerebellum [78]. Moreover, expanded ATXN1 has been shown to alter the activity and localisation of several interacting proteins associated with nuclear transport. While these proteins were not found to be recruited into pathological aggregates, their dysfunction might still contribute to disease pathogenesis [79]. In SCA2, it was found that expanded ATXN2 loses the ability to interact with A2BP1 (RNA-binding Fox-1 homologue 1), which promotes excitotoxicity [80]. In the same line, expanded ATXN2 does not stabilize regulator of G protein Signalling 8 *(RGS8) mRNA*, leading to its degradation, which might abnormally enhance metabotropic glutamate receptor 1 (mGluR1) signalling [27]. In mouse models of SCA3/MJD, the expanded ATXN3 protein interacts abnormally with ATXN2, disrupting its interaction with PABPC1. This disruption plays a significant role in disease pathogenesis [81]. In SCA7, a toxic gain-of-function of expanded ATXN7 leads to the loss of ubiquitin C-terminal hydrolase L1 (*UCHL1*) expression [82]. Finally, in SCA17, it was shown that the PolyQ tract affects the binding of TBP to the DNA and that PolyQ-expanded TBP can induce neuronal toxicity independent of its interaction with DNA [67]. Although these abnormal interactions suggest a toxic gain-of-function in the expanded polyQ protein, they could also lead to a loss-of-function. In fact, a study described that the partial loss of ATXN1 function leads to transcriptional dysregulation in mouse models, contributing to SCA1 pathogenesis [25]. Also, some studies point out that a loss-of-function of the ATXN3 protein contributes to a dysregulation of transcriptional activity, which contributes to SCA3/MJD pathogenesis [83, 84].

2) The domains and motifs present in the abnormally expanded polyQ proteins interact with other proteins and molecules in a different manner than the non-expanded protein [64, 85]. In SCA1 *Drosophila* and mouse models, it was shown that the AXH domain present in the expanded ATXN1 mediates its abnormal binding to the transcription factor Gfi-1, contributing to disease neuropathology [86]. In SCA3/MJD, expanded ATXN3 is able to sequester its interacting partners into inclusions through specific motifs, impairing their normal cellular function and contributing decisively to the disease [87]. In SCA7, a study reported that the zinc-finger domain of expanded ATXN7 sequesters ubiquitin-specific peptidase 22 (USP22) into a catalytically inactive state, impairing its normal cellular function [88]. Proteins are also subjected to posttranslational modifications (PTMs), which clearly impact the disease pathogenesis and that we recognized as a primary hallmark of PolyQ SCAs. But do PTMs alter the interaction of polyQ proteins, impacting the disease pathogenesis? In SCA1 models, it was shown that, in the expanded ATXN1, phosphorylation of S776 enhances its interaction with molecular chaperone 14-3-3, contributing to neurodegeneration [89]. In cellular models of SCA3/MJD, the SUMOylation of ATXN3 also affects its interaction network and its ability to self-assemble [90, 91].

The abnormal PolyQ expansion not only increases the propensity of PolyQ proteins to aggregate but also alters normal protein interactions and induces new ones. Abnormal protein-protein interactions triggered by PolyQ expansion may involve chaperones, RNA-binding proteins, and transcription factors, disrupting crucial cell systems and contributing to toxicity and neurodegeneration. Aggregation and abnormal interactions are closely linked, with some authors speculating that aggregation itself is a form of aberrant interaction [19].

### Posttranslational modifications

Posttranslational modifications are chemical alterations that proteins are subjected to after translation. PTMs include reversible modifications such as phosphorylation, acetylation, SUMOylation, and ubiquitination, as well as irreversible changes like proteolytic cleavage. These modifications play important roles in regulating protein structure, function, localisation, and interactions within the cell. In a disease context, including in PolyQ SCAs (Table 2), proteins may be targeted by aberrant PTMs that underly pathogenesis [92].

**Table 2:** Selected posttranslational modifications detected in PolyQ SCAs.

SCA	PTM location	PTM type	PTM outcome	Reference:
SCA1	S776	Loss of Phosphorylation	Protein stability enhancement	Chen HK, Fernandez-Funez P, Acevedo SF, Lam YC, Kaytor MD, Fernandez MH, et al. Interaction of Akt-Phosphorylated Ataxin-1 with 14-3-3 Mediates Neurodegeneration in Spinocerebellar Ataxia Type 1. Cell. 2003 May;113(4):457–68. Jorgensen ND, Andresen JM, Lagalwar S, Armstrong B, Stevens S, Byam CE, et al. Phosphorylation of ATXN1 at Ser776 in the cerebellum. J Neurochem. 2009 Jul;110(2):675–86
	Lys(16), Lys(194) Lys(610)/Lys(697) and Lys(746)	SUMOylation	Increase aggregation	Riley BE, Zoghbi HY, Orr HT. SUMOylation of the Polyglutamine Repeat Protein, Ataxin-1, Is Dependent on a Functional Nuclear Localization Signal. J Biol Chem. 2005 Jun;280(23):21942–8
		Ubiquitination		Al-Ramahi I, Lam YC, Chen HK, De Gouyon B, Zhang M, Pérez AM, et al. CHIP Protects from the Neurotoxicity of Expanded and Wild-type Ataxin-1 and Promotes Their Ubiquitination and Degradation. J Biol Chem. 2006 Sep;281(36):26714–24
SCA2		Phosphorylation	Neuroprotective	100
		Ubiquitination		Huynh DP, Nguyen DT, Pulst-Korenberg JB, Brice A, Pulst SM. Parkin is an E3 ubiquitin-ligase for normal and mutant ataxin-2 and prevents ataxin-2-induced cell death. Exp Neurol. 2007 Feb;203(2):531–41
SCA3/MJD	S12	Phosphorylation	Aggregation reduction and dendrite and synapse loss	Matos CA, Nóbrega C, Louros SR, Almeida B, Ferreiro E, Valero J, et al. Ataxin-3 phosphorylation decreases neuronal defects in spinocerebellar ataxia type 3 models. J Cell Biol. 2016 Feb 15;212(4):465–80.
	S256	Phosphorylation	Aggregation reduction	Fei E, Jia N, Zhang T, Ma X, Wang H, Liu C, et al. Phosphorylation of ataxin-3 by glycogen synthase kinase 3β at serine 256 regulates the aggregation of ataxin-3. Biochem Biophys Res Commun. 2007 Jun 1;357(2):487–92
	S236, S260, S261, S340, and S352	Phosphorylation	increases nuclear localization, protein stability and nuclear inclusion formation	Mueller T, Breuer P, Schmitt I, Walter J, Evert BO, Wüllner U. CK2-dependent phosphorylation determines cellular localization and stability of ataxin-3. Hum Mol Genet. 2009 Sep 1;18(17):3334–43. Tao RS, Fei EK, Ying Z, Wang HF, Wang GH. Casein kinase 2 interacts with and phosphorylates ataxin-3. Neurosci Bull. 2008 Oct 3;24(5):271–7
	S256	Phosphorylation	Neuroprotective/Neurotoxic	Fei E, Jia N, Zhang T, Ma X, Wang H, Liu C, et al. Phosphorylation of ataxin-3 by glycogen synthase kinase 3β at serine 256 regulates the aggregation of ataxin-3. Biochem Biophys Res Commun. 2007 Jun 1;357(2):487–92. 103.Mueller T, Breuer P, Schmitt I, Walter J, Evert BO, Wüllner U. CK2-dependent phosphorylation determines cellular localization and stability of ataxin-3. Hum Mol Genet. 2009 Sep 1;18(17):3334–43. 104.Tao RS, Fei EK, Ying Z, Wang HF, Wang GH. Casein kinase 2 interacts with and phosphorylates ataxin-3. Neurosci Bull. 2008 Oct 3;24(5):271–7
	K356	SUMOylation	Aggregation reduction	Almeida B, Abreu IA, Matos CA, Fraga JS, Fernandes S, Macedo MG, et al. SUMOylation of the brain-predominant Ataxin-3 isoform modulates its interaction with p97. Biochim Biophys Acta BBA - Mol Basis Dis. 2015 Sep 1;1852(9):1950–9
	K166	SUMOylation	Enhance protein stabilization and toxicity	hou YF, Liao SS, Luo YY, Tang JG, Wang JL, Lei LF, et al. SUMO-1 Modification on K166 of PolyQ-Expanded aTaxin-3 Strengthens Its Stability and Increases Its Cytotoxicity. PLoS ONE. 2013 Jan 31;8(1):e54214
		Ubiquitination		Matsumoto M, Yada M, Hatakeyama S, Ishimoto H, Tanimura T, Tsuji S, et al. Molecular clearance of ataxin-3 is regulated by a mammalian E4. EMBO J. 2004 Feb 11;23(3):659–69
SCA7	K257	Acetylation	Inhibits protein turnover	Mookerjee S, Papanikolaou T, Guyenet SJ, Sampath V, Lin A, Vitelli C, et al. Posttranslational Modification of Ataxin-7 at Lysine 257 Prevents Autophagy-Mediated Turnover of an N-Terminal Caspase-7 Cleavage Fragment. J Neurosci. 2009 Dec 2;29(48):15134–44
	K257	SUMOylation	Aggregation reduction	Janer A, Werner A, Takahashi-Fujigasaki J, Daret A, Fujigasaki H, Takada K, et al. SUMOylation attenuates the aggregation propensity and cellular toxicity of the polyglutamine expanded ataxin-7. Hum Mol Genet. 2010 Jan 1;19(1):181–95

1) In PolyQ SCAs, phosphorylation is the reversible PTM that has more impact on disease pathogenesis. In SCA1, the phosphorylation of ATXN1 at S776 enhances protein stability, leading to accumulation of the mutant protein and exacerbating neurodegeneration [89, 93]. In SCA2, phosphorylation of ATXN2 seems to be neuroprotective, leading to a greater degradation of the mutant protein by the ubiquitin–proteasome system (UPS) in comparison to normal ATXN2, potentially mitigating toxicity [94]. In SCA3/MJD, ATXN3 is phosphorylated at S12, reducing aggregation as well as dendrite and synapse loss [95]. Additionally, phosphorylation of ATXN3 at S256 decreases aggregation, also suggesting a protective effect. However, in this context, PolyQ-expanded ATXN3 is less phosphorylated than its wild-type counterpart [96]. On the contrary, ATXN3 phosphorylation at S236, S256, S260, S261, S340, and S352 increases nuclear localization, protein stability, and nuclear inclusion formation [97, 98].

PTMs, such as acetylation and SUMOylation, also have a significant influence over PolyQ proteins. In SCA7, acetylation of ATXN7 at the K257 site decreases protein turnover, leading to accumulation of toxic ATXN7 fragments [99]. On the other hand, SUMOylation can reduce aggregation and alleviate toxicity, as observed when ATXN3 is SUMOylated at K356 and ATXN7 at K257 [91, 100]. However, SUMOylation seems to play a dual role in SCA3/MJD, since it can enhance protein stabilization and toxicity when occurring at K166 [90]. SUMOylation of ATXN1 at different residues increases aggregation, but SUMOylation is reduced in polyQ-expanded ATXN1, suggesting the existence of a self-protective mechanism [101]. An opposite effect is exerted by enhanced oxidative stress, which increases ATXN1 SUMOylation and aggregation. The loss of phosphorylation at S776 in the ATXN1 protein, which induces toxicity [89, 93], restores the normal levels of SUMOylation in this protein [102].

Ubiquitination also emerges as a critical regulator of polyQ proteins homoeostasis, orchestrating protein degradation through the UPS, namely in the cases of ATXN1, ATXN2, and ATXN3. Ubiquitin conjugation targets the PolyQ proteins for degradation, decreasing protein levels, aggregation, and toxicity [94, 103–107]. However, how can a PTM at the same amino acid residue lead to different outcomes? One example is the phosphorylation of ATXN3 at S256 by different kinases that leads to different outcomes. While GSK 3β phosphorylation of ATXN3 at S256 seems to increase its solubility, potentially exerting protective effects, CK2 phosphorylation of ATXN3 at the same amino acid residue seems to produce neurotoxic effects by increasing ATXN3 nuclear abundance and aggregation [96–98]. This adds an additional layer of functional protein regulation, which could explain the dual role of the same PTM and might even account for the varying susceptibility of different neuronal populations and the phenomenon of selective neurodegeneration. It also raises the question of whether the dysregulation of PTMs in PolyQ SCAs results from a highly complex network where certain PTMs are central and disrupt others.

2) Proteolytic cleavage of several PolyQ SCAs proteins has been described. However, the significance of the resulting protein fragments in pathogenesis is not the same for all diseases. Several studies suggest that the formation of proteolytic ATXN3 fragments contributes to SCA3/MJD development [2]. Several studies have shown that both expanded and non-expanded ATXN3 are subjected to proteolytic cleavage. Truncated ATXN3 with an expanded PolyQ tract showed apoptotic-inducing features in COS-7 cells, which were not observed in either full-length ATXN3 or the equivalent non-expanded fragments [108]. Furthermore, ATXN3 fragments showed increased aggregation propensity and toxicity compared to the full-length protein, as evidenced in several cell and animal models [30, 108–115]. Although the toxicity of full-length ATXN3 is recognized [95, 116], some researchers consider C-terminal fragments with expanded PolyQ sequences to be more pathogenic [117, 118]. In other PolyQ SCAs, the implications of proteolytic fragments are less clear. In an SCA1 transgenic mouse model, ATXN1 generates C-terminal fragments lacking the PolyQ tract, while the expected complementary N-terminal fragments containing the polyQ tract were not detected [47]. ATXN2 undergoes cleavage, resulting in two main fragments: a 42 kDa fragment containing the polyQ region and a 70 kDa fragment lacking the polyQ stretch [119, 120]. The 42 kDa fragment with the PolyQ region was found to be enriched in brain samples from SCA2 patients, suggesting a potential involvement in SCA2 pathogenesis. Additionally, several C-terminal fragments of ATXN2, including the one with 70 kDa, were identified in SH-SY5Y cells, but their functions are still unknown [121]. In SCA6, Cav2.1 undergoes fragmentation, resulting in truncated fragments containing the C-terminal expanded PolyQ tract that have been associated with increased cell death, compared to the full-length protein [122]. Moreover, transgenic mouse models expressing truncated Cav2.1 exhibited ataxia and Purkinje cell loss, suggesting a role of these fragments in SCA6 pathogenesis [123]. Expanded ATXN7 expressed in rod photoreceptors or Purkinje cells generates a 150 kDa fragment containing the polyQ tract, which aggregates into inclusions [124]. Similarly, a soluble N-terminal fragment of 55 kDa was detected in transgenic mice expressing expanded ATXN7 and in SCA7 patient fibroblasts, but not in healthy controls, suggesting a potential involvement in pathogenesis [125, 126]. Similarly, N-terminal fragments of TBP containing the PolyQ region were detected in brain lysates from SCA17 transgenic mice, but not in wild-type animals. However, their significance in disease pathogenesis requires further investigation [67].

While there is considerable evidence highlighting the importance of proteolytic cleavage and protein fragment formation in PolyQ SCAs, it remains unclear whether specific proteolytic fragments are present in patients, what is their role in pathogenesis, and whether they could serve as therapeutic targets for PolyQ SCAs. In SCA3/MJD, some studies showed that the inhibition of ATXN3 cleavage mediated by calpains alleviated deficits in different mouse models of the disease [118, 127]. However, these questions are still unsolved and there are incongruencies between the fragments found in cell and animal models and in patients [2].

In conclusion, alterations in PTMs of PolyQ-expanded proteins are common in PolyQ SCAs. However, further studies are needed to understand their contribution to the intrinsic toxicity of these proteins and their impact on downstream pathways.

### RNA-mediated dysfunction

It is becoming increasingly clear that abnormalities in RNA processing are a common feature across many neurodegenerative diseases [128]. In PolyQ SCAs, RNAs with expanded CAG repeats may contribute to cellular pathology through two distinct RNA-triggered processes. First, the cellular toxicity may result from protein gain-of-function or loss-of-function of RNA-binding proteins (RBPs) sequestered into mutant RNA foci. Second, RNAs with expanded CAG repeats may undergo aberrant repeat-associated non-AUG (RAN) translation, which generates aggregation-prone peptides (RAN peptides) from multiple reading frames without the canonical AUG start codon [129, 130].

1) Emerging evidence suggests that, in PolyQ SCAs, the RNA itself, with its expanded CAG repeats, also plays a direct contributing role in the molecular pathogenesis of these disorders [131]. In SCA2 cellular models, it was shown that RNA toxicity leads to the disruption of ribosomal RNA processing [132]. Moreover, in an SCA2 *Drosophila* model, the expression of expanded ATXN2 with a pure CAG tract caused toxicity in the retina and nervous system. In contrast, expanded ATXN2 with CAA interruptions did not result in toxicity [29]. Similarly, in a SCA3/MJD *Drosophila* model, expanded ATXN3 with a pure CAG tract was more toxic than a similar form with CAA interruptions [133].

While it is established that expanded CAG repeat-containing RNA can be toxic, the precise mechanisms underlying this toxicity remain unclear. Which specific cellular processes are disrupted by the presence of these expanded RNA repeats, and how do they lead to cellular dysfunction and neurodegeneration? One component of RNA toxicity could be the disruption of overall RNA metabolism, including transcription, splicing, transport, and translation. In fact, there is evidence that RNA metabolism and its molecular players are affected in neurodegenerative diseases, including in PolyQ SCAs [134, 135].

2) RBPs interact dynamically with coding and non-coding RNAs, forming functional foci termed ribonucleoprotein (RNP) complexes [136]. Mutant RNA foci formation is a common feature of PolyQ SCAs, and foci number positively correlates with CAG repeat length [137]. It was found that several RBPs are sequestered into these abnormal phase-separated RNA foci, and that their cellular functions become consequently impaired. Muscleblind-like splicing regulator 1 (MBNL1) is sequestered into RNA foci containing expanded *ATXN1*, *ATXN3*, and *ATXN7* transcripts in SCA1, SCA3/MJD, and SCA7 patient fibroblasts, respectively [137, 138]. Additionally, in an SCA2 cell model, MBNL1 accumulates in RNA foci containing mutant ATXN2-antisense strand (ATXN2-AS) transcripts with expanded CUG repeats [139]. In SCA3/MJD, MBNL1 co-localization with mutant RNA foci increases in a CAG repeat length-dependent manner, and alternative splicing dysregulation is observed in vitro [137, 138].

While it seems that aberrant foci are relevant for PolyQ SCAs molecular pathogenesis, several questions are still open. For example, what is the role of specific RNP complexes, such as stress granules (SG), in these diseases? SG are membrane-less cell compartments formed in response to different stress stimuli, wherein translation factors, mRNAs, RBPs, and other proteins coalesce together. They are implicated in the regulation of translation, mRNA storage and stabilization and cell signalling, during stress. Available data concerning the abnormal properties of the expanded polyQ proteins and their involvement in stress response dysregulation strongly suggest an important role for SG in the pathogenesis of PolyQ SCAs [140]. Important SG components, such as RBPs, are sequestered to pathological inclusions, impairing the normal functioning of those proteins and the SG dynamics and function. In SCA2, the SG component TAR DNA-binding protein 43 (TDP-43) co-localizes with pathological inclusions of expanded ATXN2 [141], itself a SGs component that is also found in pathological inclusions in SCA3/MJD [142].

3) Expanded CAG RNAs can form stable hairpin structures with distinctive dsRNA segments, which are substrates for Dicer, generating small toxic RNA fragments – small CAG repeats fragments (sCAGs) – that behave as endogenous miRNA-like molecules [143, 144]. The sCAGs generated from CAG repeats in expanded ATXN1, ATXN2, and ATXN3 transcripts were detected in cellular models of SCA1, SCA2, and SCA3/MJD, respectively [140–145]

4) RAN translation was discovered in 2011 [146], and since then, it has been described in several microsatellite expansion disorders, including PolyQ SCAs [147]. RAN proteins are translated from different types of nucleotides repeat expansions and can be produced from both sense and antisense transcripts. In SCA2, experiments using cell lines and various ATXN2 constructs with mutations in all ATG trinucleotide upstream of the CAG repeats revealed that RNA translation still occurred, producing RAN PolyQ, PolyA, and PolyS peptides [148]. In SCA3/MJD, RAN translation was also observed in a cell model in which RAN polyQ and RAN polyA peptides were found exclusively in cells expressing expanded ATXN3 [149]. While the discovery of RAN translation has added complexity to the molecular pathogenesis of PolyQ SCAs, the actual presence and significance of RAN peptides in patient brains remain uncertain. In cell and animal models, RAN translation was observed; however, the evidence for the presence of RAN peptides in the *postmortem* brain tissues of PolyQ SCAs patients is nonexistent.

Although RNA-mediated dysfunction is difficult to assess in coding expansion disorders, where diverse protein and RNA toxic effects may coexist, RNA-induced toxicity seems clearly implicated in PolyQ SCAs pathogenesis. Understanding the relative contribution of the mutant sense and/or antisense deleterious RNA-dependent mechanisms to pathogenesis is crucial for the development of potential therapeutic strategies for PolyQ SCAs, particularly those that directly target expanded RNA or even expanded DNA.

## Secondary Hallmarks

### Mitochondrial dysfunction

Mitochondria are instrumental players in crucial processes that ensure cell survival, with special emphasis on energy metabolism, biosynthesis, calcium homoeostasis maintenance, modulation of reactive oxygen species (ROS) levels, and nutrient signalling; additionally, mitochondria also play a critical role on apoptosis induction [150]. Mitochondrial dysfunction may arise or be exacerbated through impairment of the mitochondrial electron transport chain (ETC), oxidative stress, and mitochondrial DNA (mtDNA) damage [151, 152]. The fact that dysfunction in mitochondria greatly impacts neuronal homoeostasis is highlighted by the existence of a positive correlation between mitochondrial dysfunction and neurodegeneration [152–154].

1) Mitochondrial ETC protein complexes have been shown to have compromised activity in cell models of SCA2 [151] and SCA3/MJD [155, 156], in various brain regions of SCA1 mouse models [157, 158], and in both patient samples and mouse models of SCA7 [159, 160]. These complexes are involved in the oxidative phosphorylation system (OXPHOS), responsible for ATP production and are a major intracellular producer of ROS. Neurons are highly dependent on oxidative phosphorylation to meet their functional energy requirements. Thus, the disruption of ETC and OXPHOS activity in PolyQ SCAs may result in insufficient energy production and exacerbated ROS generation, promoting neuronal dysfunction [152]. In fact, the mitigation of OXPHOS deficits in an SCA1 mouse model reduced the observed neurodegeneration [157]. But what mechanisms impair ETC protein complexes functions? As these proteins are encoded by mitochondrial DNA, one hypothesis is that abnormally expanded PolyQ proteins impact the transcription and translation of these genes and proteins [161, 162], or might sequester these proteins to aggregates [163], thus impacting mitochondrial activity. However, polyQ proteins are not normally found in mitochondria, nor have the pathological aggregates been detected in these organelles.

2) Several studies showed that the antioxidant system functioning is compromised in PolyQ SCAs. Under normal physiological conditions, the mitochondrial antioxidant defence mechanism counteracts the ROS levels generated during regular mitochondrial function. However, when ROS levels exceed the capacity of the antioxidant system or if the system is impaired, oxidative stress occurs, leading to cell damage [164]. The levels of antioxidant enzymes such as catalase, superoxide dismutase 2 (SOD2), and other oxidative stress markers were shown to be dysregulated in cellular models for SCA2, SCA3/MJD and SCA7 [33, 151, 165–168], in an SCA7 mouse model [169] and in SCA2, SCA3/MJD and SCA7 patient samples [170–172]. Neurons are especially vulnerable to oxidative damage, considering they require high availability of oxygen, high levels of structures susceptible to oxidative damage and a poor antioxidant defence mechanism [16, 173]. But does oxidative stress directly contribute to the pathogenesis of PolyQ SCAs? Stress conditions may exacerbate PolyQ SCAs molecular features. In fact, it was shown that oxidative stress promotes the translocation of expanded ATXN3 to the nucleus and promotes ATXN7 aggregation, both increasing toxicity [169, 174]. On the other hand, the administration of antioxidants to cellular and animals models of SCA1, SCA2, and SCA3/MJD improved neuronal integrity and delayed motor deficits in mouse models [151, 162, 175].

3) Defects in mtDNA integrity were observed in PolyQ SCAs. mtDNA encodes several genes involved in energy homeostasis, including essential subunits of the ETC protein complexes involved in OXPHOS. In SCA1, expanded ATXN1 impairs mtDNA maintenance, thereby indirectly promoting the accumulation of mtDNA damage [176]. An increase in DNA damage was reported in cell models for SCA3/MJD [165], as well as in SCA1 and SCA3/MJD mouse models [176, 177] and in SCA2 and SCA3/MJD patients’ samples [178]. These alterations may result in neuronal loss through energy deficiency and/or oxidative damage [179]. Gene supplementation of a mtDNA repair protein improved the motor phenotype of an SCA1 mouse model [176].

4) Mitochondria harbour many key factors triggering cell death pathways, namely the mitochondria-mediated caspase activation pathway of apoptosis [180]. Dysregulation of pro-apoptotic factors was observed in PolyQ SCAs, promoting neuronal dysfunction and death. In SCA3/MJD, expanded ATXN3 accumulates in mitochondria, activating the apoptotic pathway and leading to neuronal death by upregulating Bax expression and downregulating Bcl-(xL) expression [153, 181, 182]. Similar results were observed for expanded ATXN7 in cellular models of SCA7 [154].

5) Mitochondria are part of a dynamic network that undergoes fusion, fission, and morphological changes, ensuring that their integrity and number meet cellular demands and enabling the turnover of dysfunctional mitochondria [183]. Defects in mitochondrial dynamics have been detected in PolyQ SCAs, specifically in SCA1, SCA2, SCA3/MJD, and SCA7. Abnormal mitochondrial morphology was observed, and an impairment of fusion and fission dynamics has also been described [151, 160, 184]. These alterations lead to the accumulation of dysfunctional mitochondria, which in turn contribute to progressive oxidative stress and increased neuronal death [183]. Could an impairment in the degradation of defective mitochondria through mitophagy blockade contribute to disease pathogenesis? A recent study in SCA6 suggests that an impairment in mitophagy exacerbates mitochondrial dysfunction at late disease stages, contributing to disease progression [185].

All these studies indicate that mitochondrial dysfunction plays a key role in the aetiology of PolyQ SCAs. This is important, as targeting mitochondrial dysfunction in its different aspects has proven beneficial in ameliorating PolyQ SCAs phenotypes, both in vitro and in vivo.

### Dysregulation of cellular degradation systems

As with many other neurodegenerative diseases, it is well-established that PolyQ SCAs involve impairments in degradation systems, specifically the ubiquitin-proteasome system and autophagy.

#### The ubiquitin-proteasome system

The UPS is a degradation pathway involved in clearing short-lived, damaged, and misfolded nuclear and cytoplasmic proteins. UPS-mediated degradation consists of two main steps, the identification of the target substrates, through polyubiquitin chain conjugation and the proteasomal degradation by the 26S proteasome complex [186]. Upon degradation, ubiquitin units are released, and the amino acids of degraded protein are recycled. Several studies indicate the at UPS is dysfunctional in the context of PolyQ SCAs.

1) UPS components and players have been detected in PolyQ inclusions in SCA1, SCA3/MJD, SCA7 and SCA17 patients, and this sequestration may compromise the normal functioning of this pathway [39, 42, 87, 187–190]. Evidence supports that UPS and autophagy are functionally interrelated catabolic processes [191, 192] and, thus, a compensatory activity of autophagy could occur upon UPS functional impairment. In fact, it has been shown that autophagy activation protects from neurodegeneration induced by proteasome inhibition [193, 194]. On the other hand, blocking of autophagy resulted in a massive build-up of polyubiquitinated inclusions, suggesting that the UPS is not effective in degrading their components [193, 194]. Studies suggest that proteins with long PolyQ tracts are not efficiently degraded by eukaryotic proteasomes [195]. This inefficiency, combined with impaired UPS function due to the recruitment of some UPS components to inclusions, could significantly contribute to disease pathogenesis. However, the extent to which dysfunctional proteasome activity affects the overall flux of ubiquitinated proteins remains unclear. For example, the knockdown of one ATPase subunit of the 19S proteasome in mice resulted in normal brain development, without significant abnormalities, besides reduced UPS function [196].

2) Changes in the interaction between expanded PolyQ proteins and players involved in the UPS were shown to disturb the functioning of this degradation pathway. For example, it was suggested that expanded ATXN1 drives aberrant ubiquitination and UPS dysfunction, due to its altered interaction with the A1Up proteasomal component [197]. ATXN3 has been reported to play a role in UPS, as a ubiquitin protease editing polyubiquitin chains, and as a shuttler of substrates for proteasomal degradation [198–201]. The absence of ATXN3 led to an increase in the levels of ubiquitinated proteins in the brain [198]. On the other hand, expanded ATXN3 leads to a reduction of deubiquitinated proteins [202]. ATXN7 interacts with the ATPase subunit S4 of the 19S proteasomal subunit, and the stability of this interaction is inversely correlated with the length of the polyQ tract [203]. PolyQ-expanded proteins impact UPS function, but could the UPS serve as a therapeutic target for PolyQ SCAs therapy? Studies investigating the stimulation of the UPS are mainly limited to SCA3/MJD and to a lesser extent to SCA1 [204]. It was shown that the overexpression of Ub ligase C-terminus of Hsc-70-interacting protein (CHIP) confers neuroprotection by enhancing ubiquitination and degradation of expanded ATXN1 [103] and expanded ATXN3 [105, 205]. Similarly, increasing the levels of other factors related to the UPS has been shown to enhance the breakdown of expanded ATXN3, reducing neurodegeneration. However, this approach may not fully address the underlying dysfunctions in the UPS itself [106, 206–208]. Although some preclinical results are encouraging, further research is necessary to determine whether stimulating the UPS could be a viable therapeutic strategy for PolyQ SCAs.

#### Autophagy

Autophagy is a lysosomal-mediated degradation pathway that plays a major role in catalysing the turnover of large targets, including aged or damaged organelles and misfolded proteins. Autophagy occurs through three primary pathways, according to the particular lysosomal-internalization mechanism that takes place: macroautophagy (commonly named simply as autophagy), microautophagy and chaperone-mediated autophagy (CMA) [209]. Neurons critically depend on a fully working autophagic system to prevent toxic protein and dysfunctional organelle accumulation and to ensure proper neuronal function. The central role of autophagy in neurons was shown by two loss-of-function studies, where suppression of this pathway in a healthy condition caused neurodegeneration [210, 211]. The conditional knock-out of the autophagic genes *Atg5* and *Atg7* in the CNS resulted in neurodegenerative signs comparable to those observed in several neurodegenerative disorders, clearly supporting a crucial role for autophagic impairment in neurodegeneration [211, 212]. Furthermore, the knockout of *Atg7* in Purkinje cells led to motor deficits resembling the motor alterations observed in mouse models of PolyQ SCAs [213]. These findings underscore the importance of autophagy for neuronal survival and laid the foundation for understanding that disruptions in autophagy contribute to the development of neurodegenerative diseases, including PolyQ SCAs [214, 215].

1) Major autophagic players such as beclin-1 and sequestosome 1 (p62/SQSTM1) accumulate within the pathological inclusions of PolyQ SCAs [216–221]. This accumulation could limit their availability and compromise autophagy. In fact, some studies showed that there is a reduction in the levels of several autophagic proteins in SCA2 and SCA3/MJD, while others showed an accumulation of p62/SQSTM1, suggesting ineffective lysosomal degradation [211–213, 216, 217, 222]

2) At least some of the proteins causing PolyQ SCAs interact or otherwise interfere with components of the autophagic pathway. Consequently, and alterations in these interactions may contribute autophagy dysfunction. Expanded ATXN2 expression leads to an overabundance of Staufen1, which is a regulator of mTOR signalling, impairing the autophagic pathway [60, 223]. ATXN3 is a substrate for autophagy [224], and regulates autophagy by deubiquitinating Beclin-1 and protecting it from proteasomal degradation. Blocking of the ATXN3-Beclin-1 interaction results in decreased Beclin-1 levels and impaired starvation-induced autophagy [225]. Expanded ATXN7 recruits two key proteins involved in autophagy initiation to aggregates, leading to a destabilization of Unc-51-like autophagy activating kinase 1 (ULK1) and a decreased capacity for autophagy induction [226]. In SCA17, expanded TBP interacts with high mobility group box 1 protein (HMGB1), recruiting it to aggregates. This protein is an important regulator of autophagy, and its recruitment leads to a dysfunctional starvation-induced autophagy [220].

3) The role of microautophagy and CMA in the pathogenesis of PolyQ SCAs is not well established, possibly due to the limited number of studies investigating these systems in these diseases. Expression of HSC70, a key component of CMA, is reduced in cells from SCA3/MJD and SCA17 patients [227]. Additionally, HSC70 has been detected in the inclusions found in cells from SCA1 patients and in animal models [55]. Are PolyQ expanded proteins substrates for CMA clearance? Lessons from other neurodegenerative diseases could shed light on this unresolved question. Recently, CMA has emerged as a contributor to neurodegenerative processes due to its role in clearing several proteins involved in neurodegenerative diseases, such as α-synuclein, tau, amyloid precursor protein, huntingtin and TDP-43 [228–230]. Overall, studies show that expanded PolyQ proteins disrupt autophagy, which is crucial for neuronal survival. Additional support for the causative role of autophagy in PolyQ SCA neurodegeneration comes from the observation that expression of autophagic genes *ATG5*, *ATG7*, and *BECN* decreases in the human brain during normal aging [222]; this could contribute to autophagy impairment and ultimately to neurodegeneration in PolyQ SCAs, which are usually recognized as adult-onset diseases. However, therapeutic strategies targeting autophagy have shown limited efficacy. Autophagy can degrade expanded proteins in the cytoplasm; however, in SCA1, SCA3/MJD, SCA7 and SCA17, PolyQ-expanded proteins accumulate in the nucleus. Could this accumulation decrease the effectiveness of autophagy in clearing the expanded proteins? This fact may help explain why therapeutic strategies designed to enhance autophagy have failed to show efficacy in modifying disease progression during clinical trials [231].

### Consequences of transcriptional dysregulation

Precise regulation of gene expression is crucial for the development of the nervous system, and for maintaining its homoeostasis throughout life [232]. Any disruption in this regulation can affect brain development and function, potentially leading to various pathologies. Several PolyQ-containing proteins are directly or indirectly involved in transcription regulation; therefore, it is not surprising that transcriptional dysregulation is often described to be a pathological alteration involved in these diseases.

1) Several alterations in gene expression were found in animal models and *postmortem* brain tissue from patients with PolyQ SCAs [233–235]. In an SCA1 transgenic mouse model, alterations in the expression of several genes involved in glutamate signalling pathways were found in Purkinje cells [233]. In SCA2, the expression profiling of different mouse models of disease found profound alterations in genes specific or highly expressed in Purkinje cells [236, 237]. In SCA3/MJD, alterations in expression levels of genes involved in diverse pathways were found, including neuronal survival and differentiation, glutamatergic neurotransmission, intracellular calcium signalling/mobilization, mitogen-activated protein (MAP) kinase signalling, heat-shock proteins, oligodendrocyte myelinogenesis, and neuroinflammation [238–240].

But which are the driving factors underlying and contributing to these gene expression alterations? One important contributor may be the presence of protein aggregates in the nucleus that may interfere with transcription factors, sequestering them [241] [242]. In SCA3/MJD, it was shown that expanded ATXN3 aggregates sequester and inhibit cAMP response element-binding protein (CREB) binding protein (CBP). This causes the downregulation of the CREB signalling pathway, therefore affecting synaptic plasticity, axonal outgrowth, and cell survival [243–245]. In SCA7, it was reported that CBP and other proteins that regulate transcription are sequestered to expanded ATXN7 inclusions, contributing to the observed transcriptional dysregulation [246, 247]. Alterations in gene expression, which could be related to the transcriptional dysregulation were also observed in SCA2 and SCA6. In SCA2 patients’ blood samples, an upregulation of *FBXW8* levels was found. This alteration seems dependent on the accumulation of expanded ATXN2 [57]. In a cellular and in an animal model of SCA2, alterations in *Nat8l* mRNA levels were found as a selective effect of ATXN2 [248]. *NAT8L*, which catalyses the synthesis of N-acetylaspartate acid (NAA), is related to nervous system metabolism and myelination. In fact, SCA2 patients show an NAA reduction and consequent demyelination. In SCA6, the transcription factor of the α1A ribosomal subunit (α1ACT), encoded by expanded *CACNA1A* loses the ability to activate genes involved in neurite outgrowth and Purkinje cells development [57, 242–249].

2) Alterations in the normal interaction between PolyQ proteins and transcription factors have been observed when polyQ proteins are expanded. ATXN1 interacts with several transcriptional co-repressors/activators, including (olyglutamine-binding Protein 1 (PQBP-1) and Sp1 Transcription Factor (SP1) [242, 250, 251]. Alterations in these interactions due to the expanded polyQ tract may lead to dysfunctions in several pathways, such as Wnt-receptor signalling or retinoid acid signalling [252]. ATXN3 interacts with several transcriptional regulators, including histone acetyltransferase (HAT) proteins, CBP, histone deacetylases 1-3 (HDAC1-3), TBP, and forkhead box O4 (FOXO4), among others [167, 243, 244, 246, 253]. The polyQ expansion may compromise these interactions, leading to alterations in gene expression. For example, in SCA3/MJD there is a downregulation of FOXO4-mediated expression of SOD2, conditioning the response to oxidative stress [167]. ATXN7 interacts with the cone-rod homeobox (CRX) transcriptional factor, regulating gene expression in the retina. In SCA7, expanded ATXN7 suppresses CRX transactivation, inducing cone-rod dystrophy in a transgenic mouse model [235]. Also, in SCA17, expanded TBP shows decreased association with several transcription factors, inducing different neuropathological features [254, 255]. On the other hand, expanded TBP abnormally binds TFIIB, a component of the basal transcription machinery, reducing the expression of heat shock protein family B (small) member 1 (HSPB1) that is critical for neuronal survival and axonal integrity [256, 257].

3) Several epigenetic alterations have been observed in PolyQ SCAs. In normal conditions, HAT activity promotes histone acetylation to activate transcription [258]. In contrast, deacetylation of histones via HDAC renders gene promoters inaccessible to regulatory factors [259]. HDAC3 is a deacetylation enzyme that interacts with ATXN1. In an SCA1 mouse model, the deletion of a single allele of HDAC3 in Purkinje cells reverted some cerebellar and cognitive deficits. However, the complete loss of this protein was highly deleterious, with mice showing early-onset ataxia and evidence of progressive degeneration [260]. On one hand, in SCA3/MJD, expanded ATXN3 causes hypoacetylation of cerebellar H3 and H4 histones by impairing the activity of HAT [13, 261, 262]. This epigenetic alteration causes the downregulated expression of cerebellar genes required for long-term depression (LTD) induction in Purkinje neurons. On the other hand, the restoration of H3 and H4 acetylation levels with HDAC inhibitors, promoting HAT/HDAC balance, rescues LTD function of Purkinje cells. It ameliorates ataxic symptoms, preventing cytotoxicity and extending survival in different models of SCA3/MJD [238, 239, 261, 263, 264]. In SCA7, the expression of expanded ATXN7 disrupted SAGA assembly and decreased HAT activity, changing chromatin condensation in rod photoreceptors and repressing the transcription of a subset of genes [265–268]. Additionally, in SCA7, altered H3 histone acetylation impairs high-fidelity DNA repair, promoting cerebellar degeneration [269]. While these epigenetic alterations seem relevant for PolyQ SCAs pathogenesis, it is still not clear if they might constitute a therapeutic target to prevent disease progression. HDAC inhibition represented for long an emerging therapeutic target for several neurodegenerative diseases [270]. However, in PolyQs SCAs, reports are mostly limited to SCA3/MJD, with moderate improvements.

4) Alterations in microRNAs levels were observed in PolyQ SCAs, which could correlate with the onset and progression of pathology [271]. In SCA1, up-regulated miR-150 levels in cerebellar Purkinje neurons of a mouse model cause the reduction of vascular endothelial growth factor A (VEGFA), contributing to disease pathogenesis [272]. In SCA3/MJD several miRNAs were found to be altered. In the serum of SCA3/MJD patients, downregulation of miR-25, miR-29a, and miR-125b, which normally are involved in *ATXN3* expression regulation, was observed [273]. Another study, using brain samples and human induced pluripotent stem cells (iPSCs) derived from SCA3/MJD patients, found a dysregulation of other *ATXN3*-interacting miRNAs, such as miR-9, miR-181a, and miR-494, which may additionally contribute to transcriptional dysregulation, and consequently to disease progression [274]. In an SCA7 mouse model, lower levels of miR-124 were detected, contributing to the increased abundance of *ATXN7* transcripts. These low levels also affected the abundance of other targets of miR-124, including some involved in transcriptional regulation in the cerebellum or in the control of retinal differentiation [275, 276]. In an SCA17 cellular model, a downregulation of miR-29a/b was observed, and its interaction with the β-site amyloid precursor protein-cleaving enzyme 1 (BACE1) could be part of a molecular mechanism leading to neuronal cell death [277]. Given that alterations in miRNA levels are observed in the serum of PolyQ SCAs patients, could miRNAs be established as biomarkers for tracking disease progression? If changes in miRNA expression can be detected before clinical symptoms appear, they would constitute valuable markers for disease progression [278]. In SCA7 patients’ plasma samples, 71 differentially expressed miRNAs were found. From these, four possessed diagnostic value to discriminate between healthy controls and patients (let-7a-5p, let7e-5p, miR-18a-5p, and miR-30b-5p) [279].

Transcriptional dysregulation emerges as an important molecular hallmark of PolyQ SCAs. Changes in transcription may contribute to disease pathogenesis through several mechanisms, including sequestration of transcriptional machinery components, widespread up- or downregulation of gene expression, alteration in epigenetic modifications, and dysregulated microRNA expression. However, several questions remain unanswered, including the potential utility of epigenetic and microRNA alterations as therapeutic targets and biomarkers for disease progression.

### Defects in signal transmission

The ability of the nervous system to transfer information within a network of neurons is vital for a balanced functioning of vertebrate organism, as each neuron may communicate with hundreds of thousands of other neurons [280]. Perturbations within the structure and function of the synapse, referred to as synaptic dysfunction, can lead to alterations in neurotransmission [281]. Moreover, axonopathy can also be responsible for defects in the transmission of neuronal information between neurons [282]. Both these alterations were found to be involved in the pathogenesis of several neurodegenerative diseases, including in PolyQ SCAs [283–289].

1) Synaptic dysfunction is observed across PolyQ SCAs. Studies have shown that, in these diseases, Purkinje cell firing is impaired, Purkinje cell dendritic arborization complexity is reduced, and expression and activity of some neurotransmitter receptors are altered [290–292]. In SCA1 mouse models, it was found that Purkinje cell firing rate is disturbed, and that the pacemaker firing rate of Purkinje cells is altered upon the onset of the disease [293, 294]. As mentioned above, in a mouse model of SCA1, gene expression alterations were observed in glutamate signalling pathways in Purkinje cells, which might contribute to synaptic dysfunction, cell degeneration and defects in neuronal information transmission [233]. In animal models of SCA2 and of SCA3/MJD, defects in Purkinje cell firing were also observed [236, 295, 296]. Additionally, in an SCA2 mouse model, the loss of the Purkinje cell marker calbindin was reported, suggesting reduced arborization complexity in these cells [297]. In SCA3/MJD and SCA6, a reduction in dendritic arborization was reported in mouse models [236, 297]. Additionally, in SCA3/MJD patients, a significant reduction of synaptic density was observed in the cerebellum and brainstem. Importantly, synaptic density in the cerebellum and brainstem was negatively correlated with disease severity [298]. In SCA1 and SCA2 mouse models, mGluR1 abnormal activity was enhanced, and downregulating this aberrant activity ameliorated the mice’s motor phenotype [290, 292].

The above studies have mostly reported synaptic dysfunction in Purkinje cells. However, dysfunction of other cells in the brain remains unclear, raising the question of how these perturbations contribute to PolyQ SCAs manifestations. A decrease or a complete loss of Purkinje cell firing would be expected to cause loss of inhibition of excitatory deep cerebellar nuclei (DCN) neurons [299]. This would result in increased excitatory input from the DCN to motor centres, causing impairments to motor performance, specifically coordination and fine-tuning of movement [300, 301]. In SCA3/MJD, a decreased number of mGluR1 receptors at the terminals of parallel fibres was shown, which could contribute to overall neurotransmission dysfunction [302].

2) Axonal defects, axonal swelling, axonal accumulation of PolyQ-expanded proteins and perturbations in axonal transport were reported in PolyQ SCAs. In SCA3/MJD mouse models and *postmortem* brain samples from patients, the presence of expanded ATXN3 inclusions in neuronal fibres in the pyramidal tract, oculomotor nerve, facial nerve, hypoglossal nerve, and nigrostriatal tract have been reported [51, 303]. Axonal swelling was observed in Purkinje cells of SCA2 patients, while demyelination, and axonal degeneration were observed in SCA1, SCA2, SCA3/MJD and SCA6 patients [303–307]. However, an important question remains: what is the effective contribution of these axonal alterations to PolyQ SCAs pathogenesis, and to the observed clinical features? So far, axonal inclusions were only reported in SCA3/MJD. Therefore, their contribution to axonal dysfunction and consequent defects in signal transmission may be limited to this disease. In SCA2, axonopathy was positively associated with disease severity and age at onset. However, for other PolyQ SCAs no such association was observed, thus making the contribution of these defects to disease pathophysiology unclear [305, 307].

Axonal defects, decrease in Purkinje cell arborization complexity, and abnormal signalling input processing may be common features shared by all PolyQ SCAs, as seen in many other neurodegenerative diseases [308, 309].

### Calcium signalling alterations

Calcium signalling is essential for neuronal function, as excessive or insufficient Ca^2+^ levels can lead to neuronal dysfunction and death [310]. Increases in Ca^2+^ influx modulate cellular excitability and promote several Ca^2+^-dependent processes, including neurotransmitter release, synaptic plasticity, gene transcription, cell division and cell death [311]. Similarly, to other neurodegenerative disorders, different physiological Ca^2+^-dependent processes have been shown to be dysregulated in PolyQ SCAs.

1) Alterations in the expression and activation of glutamatergic receptors have been observed in PolyQ SCAs. Abnormal levels of glutamatergic receptors and/or transporters have been described in cellular and animal models of SCA1 [233, 312], SCA2 [296, 313], SCA3/MJD [302, 313, 314] and SCA6 [315], impacting intracellular Ca^2+^ levels and Ca^2+^-binding protein activation. One of the most consistently reported molecular alterations is altered levels of mGluR1 [296, 302]. mGluR1 is essential for proper cerebellar function, as mice lacking mGluR1 expression are ataxic [316]. Conversely, reducing Ca^2+^ levels by blocking mGluR1 signalling has been shown to alleviate the ataxia phenotype [317]. Abnormal mGluR1-dependent signalling has been described in SCA1 and SCA2 mouse models [290, 292, 318]. In SCA1, the role of mGluR1 varies depending on the disease stage: during the early stage, mGluR1 activity is increased, while in the mid to late stages, mGluR1 levels are reduced [292, 312, 317]. In SCA6, a decrease in mGluR1 function has been linked to the onset of ataxia, even though the causative mutation also contributes to reduced Ca^2+^ levels [319, 320].

But are glutamate levels increased in PolyQ SCAs? Glutamate-mediated toxicity has been proposed to play a role in SCA1, SCA2 and SCA3/MJD pathogenesis, as increased glutamate levels aggravate disease-associated phenotypes [292, 313]. In SCA2 and SCA3/MJD, there is evidence that an increase of glutamate levels is sufficient to exacerbate the progression of cellular toxicity [313]. Additionally, expanded protein aggregates have been shown to interact with α -amino-3-hydroxy-5-methyl-4-isoxazolepropionic acid (AMPA) receptors, N-methyl-D-aspartate (NMDA) receptors, and voltage-gated Ca^2+^ channels (VGCCs), affecting their functionality [321]. In this scenario, altered Ca^2+^ homoeostasis could be largely driven by glutamate-mediated toxicity. In line with this, preclinical evidence has indicated that reducing the activation of glutamate-activated receptors mitigates aggregate formation to some extent [62]. Similar findings showed that pharmacological blockage of NMDA receptors improves mitochondrial function in SCA2 and SCA3 iPSC-derived neurons [313].

2) Changes in intracellular Ca^2+^ levels, possibly reflect impaired Ca^2+^ homoeostasis were detected in PolyQ SCAs. Increased Ca^2+^ levels were observed in SCA1 [233, 322], SCA2 [290, 318] and SCA3/MJD [323]. In SCA6, it has been proposed that there is a reduction in Ca^2+^ levels in Purkinje cells [324]. In clinical trials, the use of Ca^2+^ blockers or stabilizers as therapy to mitigate PolyQ SCAs symptoms revealed improvement in ataxia, however, the results were moderate [311, 325–327].

Altered Ca^2+^ homeostasis may result from release from the endoplasmic reticulum due to mGluR-triggered inositol trisphosphate (IP3) receptor activation. It was shown that expanded ATXN2 and ATXN3 proteins directly interact with the IP3 receptor, leading to Ca^2+^ release, mitochondrial Ca^2+^ overload, and induction of Purkinje cell death [318, 323, 328].

Ca^2+^ levels higher than physiological concentrations cause the activation of multiple Ca^2+^-sensitive enzymes and buffers. In SCA1, SCA2 and SCA7 animal models and patients’ samples, decreased levels of Ca^2+^ buffer proteins (i.e., calbindin) were observed, which possibly contribute to increases in cytoplasmic Ca^2+^ and cytotoxicity [233, 296, 329–331]. Most of these studies focused especially on Purkinje cells, but it remains unclear what is the contribution of other cell-types to altered Ca^2+^ homoeostasis.

## End-stage Hallmarks

### Neuroinflammation

Neuroinflammation is defined as the activation of the immune system in the nervous system, in response to an inflammatory signal, and is characterised by cellular and molecular changes within the nervous tissue [332]. Oligodendrocytes and astrocytes play important roles in the inflammatory response within the CNS. However, the primary immune defence in this system is carried out by microglia, which function similarly to macrophages [333–335]. It is widely accepted that neuroinflammation is involved in the pathogenesis of neurodegenerative disease, including in PolyQ SCAs [336–338].

1) In PolyQ SCAs, inflammatory signalling pathways are altered, as well as the levels of several cytokines. In SCA3/MJD cellular models and patients brain samples, increased levels of IL-1β (Interleukin 1 beta) were detected, suggesting the involvement of inflammatory processes [339]. In samples from symptomatic and asymptomatic carriers of SCA3/MJD, eotaxin (a peptide secreted by astrocytes) had differential levels between both groups of patients [340]. In SCA6 patients, a polymorphism in the IL-1β promoter was significantly correlated with the age of onset of the disease [341]. In mouse models of SCA3/MJD, SCA6 and SCA17, an overactivation of the nuclear factor kappa B subunit 1 (NF-κB1) pathway was found [342–345]. In SCA17, an overactivation of the extracellular Signal-Regulated Kinase 2 (ERK) pathway was observed, before the manifestation of motor deficits [346]. While these results seem to point out to a dysregulation of inflammatory pathways in PolyQ SCAs, it is not clear if these alterations are specific to the CNS or widespread to the entire body. A study targeting respiratory dysfunction and neuroinflammation in a SCA7 mouse model mentions that the individuals displayed both strong breathing deficits and microglia activation, indicative of neurodegeneration and neuroinflammation [347].

2) It is established that neuronal degeneration and death occur in PolyQ SCAs [348], although there is also substantial evidence showing that glial cells are altered. Studies reported that the number of astrocytes [344, 346], Bergmann glia [335], microglia [349] and oligodendrocytes are altered in different PolyQ SCAs [338, 350]. Furthermore, the activation of astrocytes, microglia, and oligodendrocytes has been observed and is linked to disease progression in SCA1 and SCA3/MJD [351, 352]. Nonetheless, it is still unclear whether glial activation results from neuronal dysfunction or from neuronal death. In SCA1 and SCA17 mouse models, an increase in gliosis and astrocyte hypertrophy markers was observed during the presymptomatic period [346, 352]. In SCA2, it was demonstrated that the expression of mutant *ATXN2* in wild-type mice lead to an increase of inflammatory response markers [222].

3) Several studies showed that the modulation of the inflammatory pathways using small molecule compounds might constitute a therapeutic strategy for PolyQ SCAs. In SCA1, the overactivation of p38 leads to a decrease of ATXN1 levels and subsequent suppression of neurodegeneration [353]. The inhibition of the c-Jun N-terminal kinase (JNK) pathway reduces Bergmann glia inflammation, which in turn manifests in phenotypical improvements in an SCA1 mouse model [335]. Similarly, reducing the activation of the NF-κB pathway led to mitigation of neurodegeneration in mouse models of SCA3/MJD, SCA6 and SCA17 [342–345]. In SCA3/MJD, the delivery of the non-steroidal anti-inflammatory drug ibuprofen to human iPSC-derived neurons brought improvements to neuropathology markers of the disease, particularly the pro-inflammatory cytokines IL-1β and tumour necrosis factor alpha (TNFα) [354]. Also, in a SCA3/MJD cellular model, the administration of three anti-inflammatory compounds suppressed the pro-inflammatory activity of cytokines such as TNFα, IL-1β, IL6, reducing ROS levels and ATXN3 aggregation, and promoting neurite outgrowth [355]. To what extent would targeting inflammation pathways be effective in treating PolyQ SCAs? It was demonstrated that neuroinflammation manifests early during PolyQ SCAs disease onset [351, 352]. Therefore, highlighting its potential as a target for disease-modifying therapies.

While alterations in inflammatory pathways occur in PolyQ SCAs, the current mainstream perspectives on disease pathogenesis are still neuron centred. It is crucial to clarify whether inflammation plays a role in the onset and progression of these diseases, specifically whether it is a consequence of the neurodegenerative process in its later stages or if it occurs before symptoms and neuronal death.

### Neuronal loss

Several alterations in neuronal function occur during ageing [356], however, neuronal loss is a feature mostly associated with neurodegenerative diseases, including PolyQ SCAs. It is a milestone event that occurs in advanced disease stages, representing irreversible damage to the affected brain regions. Neurons have several survival mechanisms that enable them to support different cytotoxic stresses [357]. When neurons undergo continuous insults and can no longer cope with stress, neuronal function is unrepairable, and neuronal degeneration ensues. Neuronal loss is the final phase of this irreversible process, in which the neuron dies due to loss of essential functions.

1) In PolyQ SCAs, neurodegeneration and neuronal loss are region-specific [348, 358, 359]. In SCA1, SCA2 and SCA3/MJD, cerebellar and brainstem degeneration are nearly always prominent, whereas regions such as the basal ganglia and the spinal cord exhibit a variable degree of damage [307, 360–362]. SCA6 is a relatively pure cerebellar syndrome with few extracerebellar signs. Macroscopically, atrophy is confined to the cerebellum, and involvement beyond the cerebellar Purkinje neurons is much less prominent than for the other PolyQ SCAs [363, 364]. SCA7 presents considerable macroscopic changes (atrophy of the cerebellum, pons and medial cerebellar peduncle), and it is typically accompanied by profound retinal degeneration [8, 364]. SCA17 shows more widespread degeneration, with patients presenting diffuse cortical and brainstem atrophy, although the most prominent degeneration occurs in the cerebellum [365]. As the expression of expanded polyQ proteins is ubiquitous, an important question is still unanswered: how to explain the selective neuronal loss that is observed in PolyQ SCAs? One possible explanation is that specific neurons are more vulnerable to the impact of the expanded protein and to the related pathogenic events [366]. Another explanation might be related to neuron features that are specific of particular neuronal populations and which might exacerbate the toxicity of the polyQ proteins, such as differences in molecular interactions or PTMs [367].

2) In PolyQ SCAs, studies have shown that neuronal loss begins before symptoms appear. The classical analysis of *postmortem* brain tissue from patients is valuable for the evaluation of the areas where neuronal loss occurs, however, they are most likely to be representative of the end-stage of the disease since cell death occurs over many years. Recent studies in SCA1, SCA2, SCA3/MJD, SCA6 and SCA7, using imaging tools and data revealed signs of neurodegeneration in several regions of the brain that correlated with initial disease symptoms and with the disease severity [368–372]. But, by which mechanisms is neuronal death occurring in PolyQ SCAs? There are a number of recognised ways by which neuronal cells can die, including apoptosis, necrosis, and autophagic cell death [373, 374]. However, it is not clear which type of cell death mechanism occurs in neuronal subpopulations in PolyQ SCAs. In other neurodegenerative diseases, apoptosis is the main driver of neuronal loss. The process could be triggered as: a ‘defence mechanism’– apoptosis is induced to remove damaged or dysfunctional neurons; or ‘death by mistake’– apoptosis is induced because of inflicted damage, such as genetic mutations, and transcriptional defects [375]. This could also be the case for PolyQ SCAs. For example, it was shown that expanded ATXN3 interaction with the DNA strand break repair enzyme polynucleotide kinase 3’-phosphatase (PNKP) inactivates its function, leading to the accumulation of DNA damage, therefore triggering apoptosis [376]. In SCA7 models, it was shown that the loss of photoreceptor neurons is caused by upregulation of apoptosis-inducing factor (AIF) and leucocyte elastase inhibitor (LEI/L-DNase II), and that cerebellar neuron loss is triggered by mitochondria-mediated apoptosis [154].

3) In most PolyQ SCAs, the cerebellum is the region most severely impacted. In SCA1, SCA2, SCA6, SCA7 and SCA17, Purkinje cells seem to be the neuronal cells most affected [377–380]. However, in SCA3/MJD these cells seem to be spared [1, 13], although some studies report moderate loss of Purkinje cells in some SCA3/MJD patients [381, 382]. But what accounts for the selective vulnerability of Purkinje cells? One hypothesis is that Purkinje cells progressively degenerate due to a decreasing ability to regulate intracellular Ca^2+^ levels [301, 315]. As already discussed, there is now evidence for a role of Ca^2+^ dysregulation in PolyQ SCAs pathogenesis. This hypothesis is quite straightforward for SCA6, as the causative mutation impacts Ca^2+^ channels, and Purkinje neurons are the most affected cells [383]. In SCA2, studies from a transgenic mouse model also support this hypothesis, as the disease phenotype is exacerbated by an increase in Ca^2+^ concentrations due to an increase in mGluR1 signalling and to a release of intracellular Ca^2+^ mediated by IP3 receptor 1 (IP3R1) [290]. As IP3R1 is uniquely expressed within Purkinje neurons, this could explain their increased vulnerability. Another hypothesis for the increased vulnerability of Purkinje neurons may lie in their unique structure, with a large cell body, long projecting axons and an extensive dendritic tree [384]. Dendritic arborization is regulated (among other things) by mitochondrial fission and transport [385]. As already discussed, there are extensive alterations in mitochondrial function that underlie PolyQ SCAs pathogenesis, which may contribute to the increased vulnerability of Purkinje cells to degeneration [184]. An additional hypothesis might be related to a deficient trophic support of their connecting cells, which might increase their susceptibility to degenerate [126].

Neuronal loss stands as an irreversible end-stage hallmark of PolyQ SCAs. The region-specific nature of neurodegeneration in these diseases, particularly within the cerebellum, underscores the complex interplay of factors contributing to selective vulnerability. While the primary hallmarks set the stage, the contribution of multiple stressors and dysfunctional pathways forms a deleterious network of events that leads to neuronal dysfunction and, ultimately, irreversible neuronal loss.

## Concluding remarks and future perspectives

Our main goal with this review article was to provide a framework of the molecular events that underlie the pathogenesis of PolyQ SCAs. We expect that this set of eleven hallmarks we identify and discuss serves as a platform for understanding the molecular mechanisms involved in these diseases and as a support for the current and future efforts in developing therapies [6, 13]. The organization of the hallmarks into primary, secondary, and end-stage categories tries to delineate a possible cascade of events spanning the transcription of the expanded gene until the neuronal death observed in PolyQ SCAs patients. But of course, current and future knowledge can provide different possibilities or hallmarks for these diseases. For us, it is crucial to set a starting point to further discuss and elucidate PolyQ SCAs pathogenesis.

It is important to stress that all these hallmarks are strongly interconnected, as aggregation may promote or result from abnormal interactions, and Ca^2+^ dysregulation may be strongly connected with mitochondrial dysfunction and defects in signal transmission, for example. Thus, the categorization we made serves mainly to give some order to the complexity of events that have been described to underlie PolyQ SCAs molecular pathogenesis. The primary hallmarks focus on the direct consequences of the presence of an expanded CAG repeat on the causative genes, emphasizing its importance to protein aggregation and other changes directly related to the expanded protein, but also its role on the known RNA toxicity. The secondary hallmarks identify the cellular pathways and organelles affected, including mitochondrial dysfunction, impaired cellular degradation systems, transcriptional dysregulation, disturbances in Ca^2+^ homoeostasis, and defects in signal transmission. The end-stage hallmarks, neuroinflammation and neuronal death, represent the conclusion of all the previous events, highlighting features that ultimately lead to the clinical manifestations observed in PolyQ SCAs.

The understanding of PolyQ SCAs molecular pathogenesis is far from complete. The identification of these hallmarks can serve as a starting point to the investigation of other mechanisms that may play crucial roles in disease pathogenesis. For example, epigenetic alterations could shed light on new levels of regulation that influence development and progression of PolyQ SCAs. Alterations in RNA granule dynamics may have constitute a link between altered protein expression, RNA toxicity and defects in signal transmission [140, 386]. Additionally, RBPs, which are increasingly recognized as key players in several neurodegenerative diseases, may also be revealed to be widely dysfunctional in the brain and constitute promising molecular targets for therapeutics [134]. Research efforts may establish novel mechanisms as new hallmarks of PolyQ SCAs, constituting essential steppingstones in the way to advance our understanding of these diseases.
